# An evaluation of the recognised systemic inflammatory biomarkers of chronic sub-optimal inflammation provides evidence for inflammageing (IFA) during multiple sclerosis (MS)

**DOI:** 10.1186/s12979-021-00225-0

**Published:** 2021-04-14

**Authors:** Christopher Bolton

**Affiliations:** Solupharm Limited UK, Monarch House, Bristol, BS3 2BX UK

**Keywords:** Biomarker, multiple sclerosis, immune tolerance, immunosenescence, inflammageing, premature immune ageing

## Abstract

The pathogenesis of the human demyelinating disorder multiple sclerosis (MS) involves the loss of immune tolerance to self-neuroantigens. A deterioration in immune tolerance is linked to inherent immune ageing, or immunosenescence (ISC). Previous work by the author has confirmed the presence of ISC during MS. Moreover, evidence verified a prematurely aged immune system that may change the frequency and profile of MS through an altered decline in immune tolerance. Immune ageing is closely linked to a chronic systemic sub-optimal inflammation, termed inflammageing (IFA), which disrupts the efficiency of immune tolerance by varying the dynamics of ISC that includes accelerated changes to the immune system over time. Therefore, a shifting deterioration in immunological tolerance may evolve during MS through adversely-scheduled effects of IFA on ISC. However, there is, to date, no collective proof of ongoing IFA during MS. The Review addresses the constraint and provides a systematic critique of compelling evidence, through appraisal of IFA-related biomarker studies, to support the occurrence of a sub-optimal inflammation during MS. The findings justify further work to unequivocally demonstrate IFA in MS and provide additional insight into the complex pathology and developing epidemiology of the disease.

## Introduction

### Immunological tolerance, autoimmunity and the epidemiology of autoimmune disease

An essential objective of the immune system is to differentiate self (auto) from non-self (non-auto) via the acquisition and maintenance of immunological tolerance which directs host defences towards the neutralisation of the potentially harmful effects of foreign antigens. The concept of immune tolerance emerged at the turn of the 20^th^ century through use of the term *horror autotoxicus*, by Paul Ehrlich, to describe the ability of an organism to distinguish self from non-self and thereby avoid injurious autoimmune reactions [1]. The notion of tolerance became accepted during the 1960s and successive theoretical and practical contributions from several distinguished scientists eventually revealed the basic mechanisms involved and the substantial importance of their findings [2, 3]. For example, Bretscher and Cohn [4] offered criteria to fulfil the theory of self-/non-self-discrimination and tolerance to auto-antigens while the practical significance of immunological tolerance was subsequently expounded by Brent [5] and referred to as ‘the holy grail of transplantation research.’

The origins of immunological tolerance incorporates a central T cell/B cell response originating in primary lymphoid organs and a separate T cell-biased reaction within peripheral tissues that prevents autoimmune events. However, there are occasions when the intricate regulatory mechanisms that maintain immunotolerance falter and, despite repeated efforts to restore equilibrium, allow biomolecular auto-antigens to become misguided targets for attack by an essentially protective but misaligned immune system. A persistent, developing and dysregulated loss of immunological self-tolerance creates an immune hyperresponsiveness and provokes pathological autoimmune reactions that feature a predominant and chronic inflammation which is sustained by thymus-derived T lymphocytes, haematopoietic cells of the monocyte-macrophage lineage and mediators that cause tissue destruction.

### Classification and epidemiology of autoimmune diseases

Unquestionably, long-term inflammatory diseases with a previously undefined autoimmune aetiology, such as rheumatoid arthritis (RA) and systemic lupus erythematosus (SLE), were noticeably present well before the knowledge and demonstration of tolerance or the understanding of immunological self and non-self [6–9]. Beneficially, the theories and observations that emerged around the acquisition and loss of immune tolerance supported a fundamental characterisation of human diseases with a recognised autoimmune profile as either tissue-specific, and involving distinguishable autoantigens, such as Type 1 diabetes, or systemic, which includes the inflammatory arthritides, and where the distribution of autoantigen is widespread [10–12].

However, there remained a lack of quantitative information on the disorders that qualified for inclusion under the diagnostic title of autoimmune disease. Indeed, and despite the acknowledged existence of autoimmune illnesses prompting the development of treatments [13], there was a persisting lack of definitive data on either incidence or prevalence of disease until the latter half of the 20^th^ century. One particular and serious concern arising from a retrospective analysis of earlier studies viewed alongside more recent epidemiological records is an undeniable and confirmed global upsurge, over the last 30 years, in the proportion and number of individuals with a diagnosed autoimmune condition [14–16]. Moreover, estimates of future global mean or period life expectancy have noticeably increased during the current millennium [17] highlighting longevity as central to the rise in incidence and prevalence.

Reports estimate that up to 10% of the world’s population experience autoimmune-associated symptoms. In detail, there has been a dramatic rise in diseases recognised as having either a definitive or inferred autoimmune pathology which includes a noticeable increase in the female-to-male ratio [18, 19]. Furthermore, a rapid escalation in the incidence of autoimmune disorders is predicted to occur over the next few decades that will undoubtedly have an important largescale effect on the provision of worldwide health care [20]. In addition, there is recognition that diseases with an autoimmune aetiology have become a major cause of morbidity and mortality.

### The decline of immune tolerance with immune ageing

Loss of immune tolerance with the development of autoimmune disease is a complex process originating from an inability to maintain immunological self-recognition through the failure of many immune cell-surface checkpoint proteins, such as glycoprotein A repetition predominant (GARP), that are under genetic and molecular regulation [21–23]. Although tolerance checkpoint malfunction appears to adopt a stochastic course there is evidence that the biological ageing process and, specifically, immune ageing compromises the network of molecular safeguards which results in the development of self-reactive immune cells and background autoimmunity [24, 25]. Immune ageing, termed immunosenescence (ISC) by Walford during the 1960s and more recently named senescent immune remodelling or immunopause, is generally defined as a gradual and subjective decline of the immune system and host defence mechanisms [26–28]. More precise observation confirms ISC to be an inherent and natural remodelling process that is genotype-dependent and sensitive to change following various internal or external environmental exposures. In particular, there is a progressive, multi-directional immunological restructuring of the innate and adaptive immune systems which profoundly affects several aspects of immunity including the development of immune tolerance and predisposition to age-related disease [29–35].

**Fig 1:**
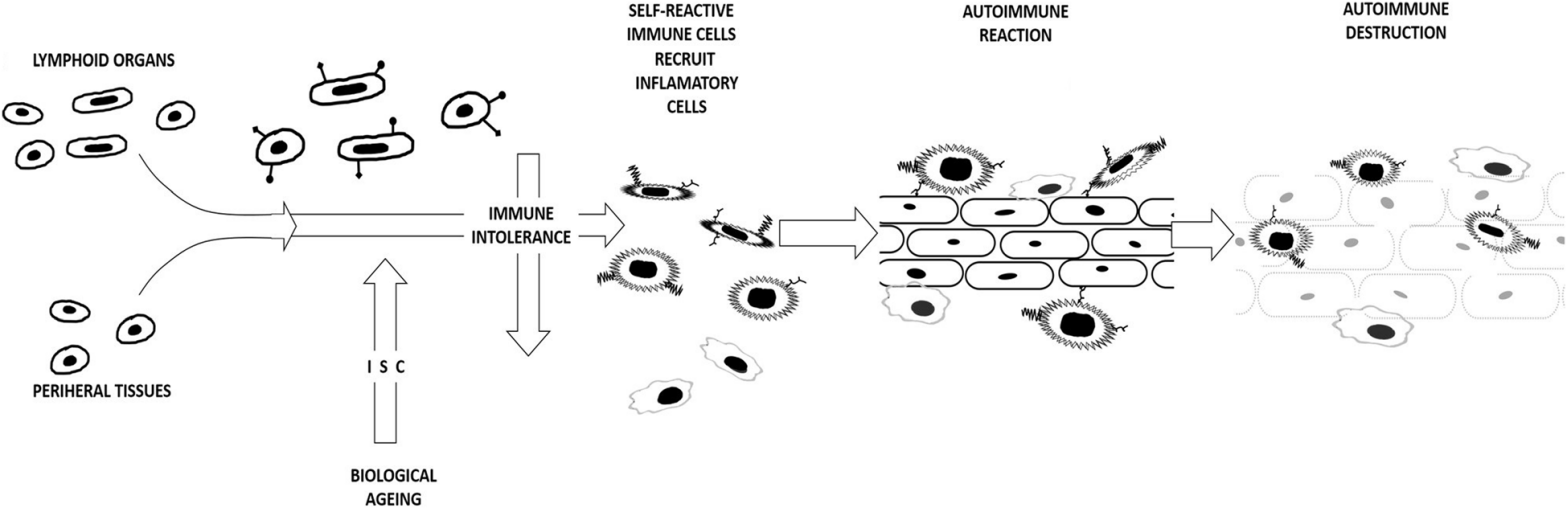
Loss of immune tolerance in an ageing immune system. T cells /B cells  in primary lymphoid organs plus peripheral T cells  express tolerance checkpoint proteins . Biological ageing-associated ISC causes a decline  in immune tolerance. Self-reactive immune cells develop  and recruit inflammatory cells . Tissue-dependent autoimmune reactions and destructive mechanisms evolve

Age-linked reorganisation of immunity manifests as quantitative and functional alterations to the essential cellular and humoral constituents of the immune system which impinge more on adaptive than innate mechanisms [36, 37]. ISC-related changes to the innate immune system include a fall in dendritic cell numbers and a reduction in the proliferation and cytotoxic activity of natural killer cells. The production of reactive oxygen species is depleted in neutrophils and also macrophages that, in addition, lose phagocytic and chemotactic capacity and the potential for cytokine generation [31, 38]. Adaptive immune alterations appear more extensive with variations in B cell numbers and B cell-dependent antibody responses, thymic involution, altered T cell subset production and function that impinges memory lymphocyte output, plus altered stem cell progenitor production and disrupted signal transduction and co-stimulatory circuits [39–41]. An appreciation of the substantial immunological changes that occur during ISC and which particularly affect immune tolerance has improved understanding of the prominent effects immune ageing has on the incidence and severity of and the susceptibility to autoimmune disease [42, 43] (Figure 1).

## Multiple sclerosis (MS)

### Deteriorating immunotolerance during MS

One disease in which a loss of immune tolerance is a prominent irregularity is the autoimmune-associated human central nervous system (CNS) disorder MS. Immunological failure to discriminate self from non-self together with chronic CNS inflammation and untimely alterations to adaptive immunity are key facets that predominate despite conjecture over the precise involvement of the immune system in the disease [44]. In particular and fundamentally, there is a primary breakdown of peripheral T cell tolerance to myelin-associated antigens preceding an enduring, immune cell-driven inflammatory pathology that is responsible for the characteristic demyelination and neurodegeneration which causes gradual and varying disability [45–49].

Interestingly, the CNS was, for many years, considered an immunologically privileged site principally because of a restrictive neurovasculature and absence of lymphatic vessels that supported an inherent ability to uphold immune tolerance and, therefore, resist the instigation of a local immune response [50]. However, the original view was questioned and recent studies employing innovative methods, including live imaging techniques, has redefined the immunological relationship between the CNS and periphery. For example, there is strong evidence for specific interaction between brain lymphatics and peripheral lymphoid tissues, together with lymphocyte subset recruitment and resident mononuclear phagocytic microglia participation, that confirms the CNS is not exclusively privileged but subject to immunological scrutiny that, if disturbed, may threaten central tolerance and contribute to the development of neurodegenerative disease [51–54].

Therefore, immunological responsiveness in CNS tissues provides an opportunity for central immune tolerance to be challenged and autoimmune reactions to develop following a requisite initial antigenic sensitisation in the peripheral lymphatics [52]. In addition, the undeniable presence of a destabilised immune tolerance in the periphery and CNS during MS will, because of the chronicity of the condition, be overlaid by ISC-linked variations that alters adaptive immunity, at both sites, with two-way effects on immune function through the course of disease.

### The history, diagnostic pathology, inherent causative links and treatment of MS

#### Historical descriptions

A decline in immune tolerance coupled with the consequences of an ageing immune system will have a cumulative and enduring effect on the development and progression of diseases with an autoimmune aetiology that includes MS. However, and typical of many autoimmune conditions, MS has an emerging, progressive and distinctive pathophysiology with typical clinical profiles that allows ultimate diagnosis of a neurodegenerative CNS disease. Historically, the first medical account of a neurological condition with MS-like symptoms was written by Augustus d’Este during the first quarter of the nineteenth century followed, during the late 1830s, by independent histopathological descriptions of the disease by Carswell and Cruveilhier [55, 56]. During the mid-nineteenth century the French neurologist, Jean-Martin Charcot, improved knowledge of the disorder through detailed histological analysis of post-mortem CNS tissues from MS patients. Furthermore, Charcot detailed diagnostic measures and suggested causative factors [57, 58].

#### Diagnosis and pathology

The intervening decades have provided opportunities for considerable improvements in diagnosis and major advances in understanding the structural and functional changes associated with the disease at cellular and mechanistic levels. For example, the destruction of myelin and the loss of oligodendrocytes at axonal sites is the consequence of events that recruit haematopoietic cells to form perivascular lesions which coalesce to create plaques and, together with resident glia, generate a destructive inflammatory environment in areas of CNS white matter. Magnetic resonance imaging techniques are routinely used to monitor plaque development and recent improvements, including magnetisation transfer, detects demyelinating events that are primarily responsible for MS symptoms which range from visual disturbances and ataxia through to limb weakness and paralysis [59]. Neurological signs vary according to the profile of disease which, from onset, may be either relapsing-remitting that develops into a secondary progressive condition or a more relentless primary progressive disorder. Immune cell-mediated inflammation is a predominant feature of relapsing-remitting MS but is less obvious in the chronic forms of disease which are more characterised by pronounced neurodegenerative events [60]. Unmistakably, and regardless of the disease phenotype, most MS patients experience a worsening of symptoms that occur over time and alongside a shifting biological environment shaped by increasing age.

#### Genetic susceptibility and defective immune tolerance

MS is an idiopathic, age-related disease and, despite the remarkable advances in our understanding of the condition plus the combined years of research dedicated to furthering knowledge, a definitive cause remains elusive. However, there is general agreement that a major influence on the autoimmune aetiology of the condition is a close connection with the genetics determining predilection for disease [12]. Indeed, a series of important genetic determinants linked to immune function exist within the major histocompatibility complex (MHC) but novel genome-wide association studies have identified many non-MHC loci related to MS susceptibility [60, 61]. A major outcome of the investigations strongly indicates a fundamental basis for MS, and indeed other autoimmune-based diseases, resides in the polygenic inheritance of genes that confer susceptibility which, in particular, includes a possible genetic link that allows a propensity towards immune tolerance failure and pathogenically-allied immune cell populations [62–66].

#### Therapies

Undoubtedly, the complex, chronic pathology associated with MS and, to date, an incomplete understanding of disease causation have, as typical of many other autoimmune conditions, frustrated a cure and delayed advances in genuinely effective therapies. Nevertheless, the past 30 years have witnessed important advances in the treatment of MS with the development of disease modifying therapies, symptomatic treatments and through attempts to redress immunological tolerance. For example, there are injectable agents including interferon-β and glatiramer acetate (Cop-1), oral drugs such as dimethyl fumarate and teriflunomide in addition to the humanised monoclonal antibodies, natalizumab and alemtuzumab [67]. Autologous hematopoietic stem cell transplantation alters immunotolerance through measurable effects on immune function and provides some lasting benefit over the clinical disease course [49]. However, managing the symptoms of progressive disease has proved more challenging with many candidate drugs, including the neuroprotective agents amiloride and riluzole, the monoclonal antibody, ocrelizumab, and other immune cell-targeted therapies, at present, unavailable for prescription while undergoing pre-clinical trials [68, 69].

#### Contemporary aetiological theories

Current progress towards new therapies are based on innovative methods that study brain networks from structural and functional imaging, evaluate immunological-related risk variants that are single nucleotide polymorphisms and assess B cell-targeted small-molecules which all derive from a number of concepts that have considered the basis of disease [66, 70]. For example, several theories on the origins of MS have been proposed including an unproven association with the measles virus, a fundamental deficiency in vitamin D and an, as yet, unsubstantiated link to the Epstein-Barr virus or viral infection which is considered causal in other autoimmune-related conditions including RA and SLE [71–76]. More recently specific bacterial pathogens have been linked to the development of neurodegenerative disorders, including MS, which has led to a multiple hit hypothesis that proposes genetic and environmental factors together with infectious agents collectively contribute to disease aetiology [77].

## The dynamics of and influences on ISC in MS

While work continues to improve the therapy for all MS phenotypes and the search for a definitive cause goes on there is a drive to understand the mechanisms underlying disease pathology through techniques including quantitative imaging and proteomics and via world-wide partnerships that encourage research and knowledge transfer [78–80]. We recently published a Review as part of the efforts to understand the immunological processes ongoing during MS [81]. Collective evidence confirmed, not unexpectedly, the inherent presence of an ageing adaptive immune system in the disease. Moreover, and unpredictably, the Review discovered and collated results from several studies to strongly indicate premature ISC over the course of the condition. We have suggested the emergence and verifiable presence of premature ageing of the adaptive immune system during the pathogenesis of MS may effect epidemiological changes and underlie the acknowledged rise in the number of adults and juveniles diagnosed with the disorder [81]. However, the mechanisms that control immune ageing and, in particular, may accelerate ISC during MS are unknown.

### ISC and an association with chronic, suboptimal inflammation in autoimmune disease

Foreseeable and untimely ISC are becoming important recognised features of other diseases with an autoimmune profile including RA, SLE and psoriasis [82–84]. Furthermore, the link between autoimmunity and the dynamics of ISC appear closely associated with a chronic and systemic low-grade inflammation, originally termed inflammageing (or inflamm-aging) (IFA) [85], which, in particular, impacts on adaptive immunity and ultimately the effectiveness of immunological tolerance through alterations in the rate of immune ageing [32, 34, 86]. Indeed, there is compelling evidence from additional studies that IFA occurs coincidently with a premature state of ISC and therefore has the potential to change the course of autoimmune diseases and, as recently indicated by us, the progression of MS [81, 87–89]. Also, and by extrapolation, there is the possibility an accelerated IFA occurs concurrently with inappropriate ageing of the immune system.

**Fig. 2:**
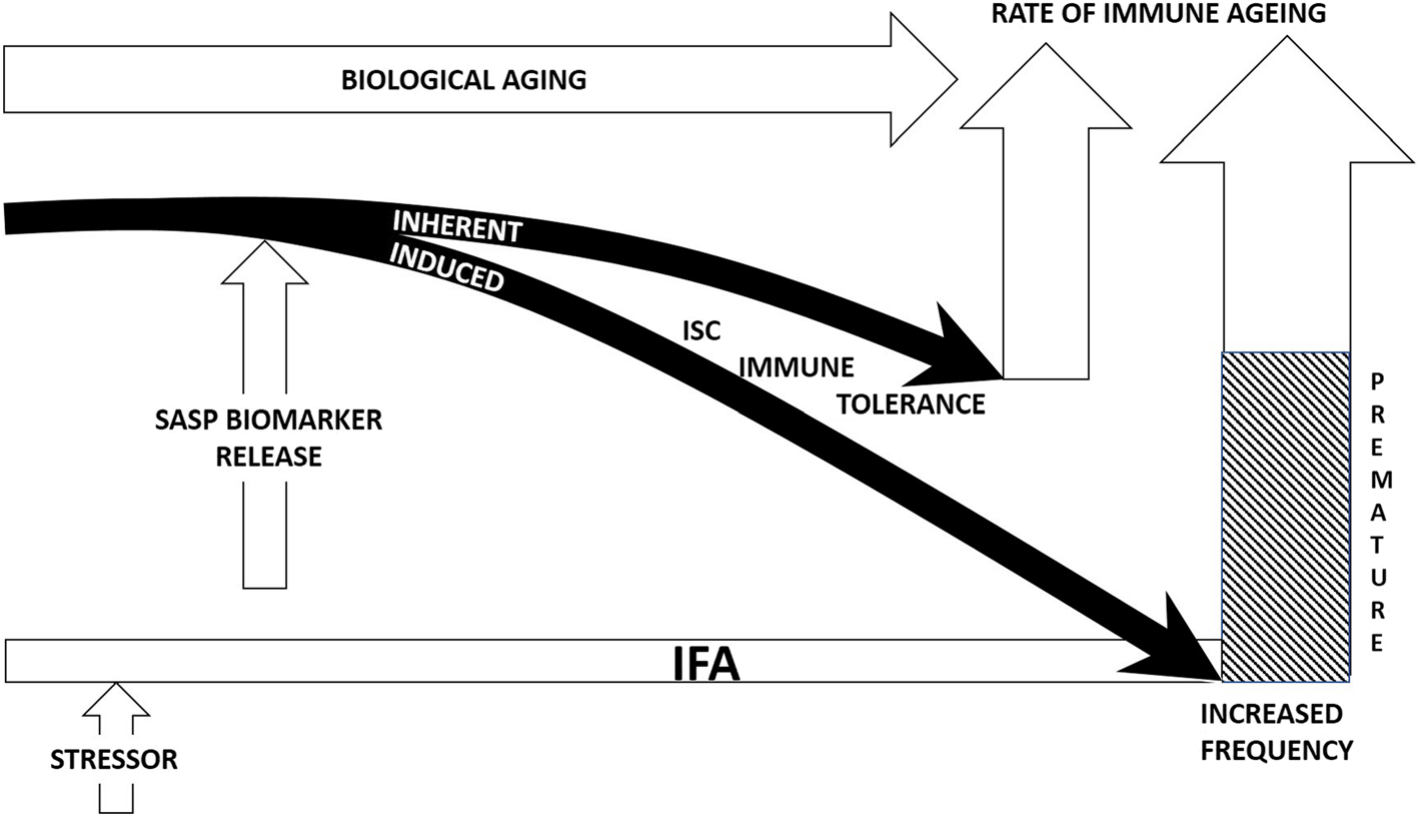
The effects of stressor-induced IFA on the dynamics of ISC and immune tolerance. Inherent ISC, as a consequence of biological ageing, influences the rate of immune ageing and immune tolerance. Stressor-activated IFA stimulates SASP release and prematurely accelerates ISC with a decline in immune tolerance and an increased frequency of autoimmune disease.

Increased understanding of the strong inter-relationship between ISC and IFA now regards ongoing sub-optimal, asymptomatic inflammation as a cornerstone of the biological ageing process (Figure 2). An initial trigger for IFA may include stressors that incorporate long-term multi-antigenic exposure in the presence or absence of a sub-clinical viral infection which, in particular, has focussed on the persistent presence of the theorised candidate cytomegalovirus (human β-herpesvirus 5) that forms part of the human β-herpesvirus family [85, 90–95] The development of IFA encompasses tissue-destructive and -restorative mechanisms that are orchestrated, at least in part and at the cellular level, by a senescence-associated secretory phenotype, or SASP, comprised of various factors including cytokines, chemokines and growth mediators which persist on the background of ISC [96–98]. Hence, there is continuous remodelling of tissues but an imbalance between damaging and reparative systems prevails because ongoing ISC has irretrievably evolved beyond regulation within the environment of progressive immune ageing. The consequence is a destructive chronic sub-optimal inflammation which causes targeted peripheral dysfunction and central disruption that may include untimely immune ageing and CNS degenerative disease [99, 100].

Therefore, IFA is viewed as an essential mainspring of practically every major age-related inflammatory-associated disorder, including RA and type-2 diabetes and, specifically, a plausible component of the enduring neuropathology that typifies MS. [101–103]. Moreover, IFA may directly affect the development of the disease through actions on ISC and, in particular, by increasing the rate of immune ageing (Figure 2). However, and to date, there is no collective evidence to support the presence of IFA during MS. Furthermore, without confirmation of IFA involvement in the pathogenesis of MS a connection with ISC and, moreover, untimely immune ageing cannot be made.

## Objectives and proposals

The purpose of the Review is to seek evidence for the presence of IFA during MS through the collective appraisal of studies that have quantitated biomarkers of low-grade inflammation. Information supporting the occurrence of IFA will offer a broad mechanism that has been shown to impact on immunological tolerance via ISC and, specifically, premature immune ageing which is notably evident during MS development and, as suggested by us, of relevance to the changing epidemiology of demyelinating disorders [81, 105].

## Identification and quantitation of IFA-correlated biomarkers

### Acknowledged biomarkers of IFA

The broad definition of a biological marker, or biomarker, is an empirically quantifiable indicator of a normal biological process or pathological state [106]. Increased detection of a biomarker suggests exposure of a biological system to harmful influences that threaten physiological, biochemical or molecular actions. IFA is linked to a cycle of pathophysiological changes, tissue damage, mounting impairment of cellular and molecular function with a loss of homeostasis that is perpetuated by altered levels of several factors including acute phase constituents and an assortment of cytokines and interleukin (IL)-related molecules [107]. There is a critical need for a clear and unanimously approved description of the features that constitute IFA in health and with the onset and progression of age-related disease. Recent developments have highlighted several putative biomarkers of IFA including microRNAs that regulate gene expression and pathways linked to chronic low-level inflammation and senescence plus an altered cluster of differentiation (CD) 4^+^/CD8^+^ ratio which we have shown to be associated with immune ageing in MS [81, 102]. Encouragingly, the persistent and frequently extreme circulating presence of inflammatory mediators associated with IFA has resulted in a more precise classification, selection and accreditation of several biomarkers that characterise the ongoing sub-optimal inflammation and related immune ageing that invariably accompanies diseases with an unbalanced immunotolerance and an autoimmune aetiology [92, 104, 108]. Hence, there is, to date, no single, specific indicator of IFA but instead a jointly classified collection of acknowledged biomarkers that include the acute phase constituent C-reactive protein (CRP), the cytokine tumor necrosis factor-α (TNF-α), IL-6 and IL-10 plus IL-1 receptor antagonist (Ra).

### Developing and refining the measurement of biomarkers that denote IFA

The elaborate pathology of MS occurs along with the variable production of many cytokines that are generated by an interactive network incorporating cellular constituents of the systemic immune system and resident inflammatory cells of the CNS [109]. A general observation from a recent and comprehensive meta-analysis has indicated that quantitation of cytokines in blood and CSF sampled from MS patients may be useful biomarkers of the disease [110]. Fundamental to the Review is the specific and joint appraisal of data measuring the concentrations of recognised biomarkers of IFA during MS in order to assess the potential for an enduring low-grade inflammation that may vary ISC, deregulate immune tolerance and alter the incidence and pathogenesis of disease. Therefore, an appreciation of pre-analytical procedures and methodologies used to quantitate and classify the inflammatory indicators is essential to aid critical scrutiny of results and conclude on the prospect of ongoing IFA during MS.

For example, systematic study of the literature has provided an understanding that separate, repeated and objective measurement of each inflammatory mediator by increasingly accurate quantitative techniques, quality control and proficiency testing procedures has allowed individual profiles to become the jointly approved biomarkers of altered immune conditions that encompass IFA [111–113]. In addition, there has been an increasing realisation that the established immune indicators, incorporating those linked to low-grade inflammation, do not function autonomously but operate as an interdependent network and therefore should be quantitated simultaneously which has promoted the use of single sample, multiple biomarker analysis [114].

Specifically, quantitation of the 5 approved inflammatory biomarkers of IFA in blood- and cerebrospinal fluid (CSF)-derived samples from MS patients and controls has progressed over the last 20 years and benefited from the improvements in analytical practices. Consequently, the intrinsic difficulties in precisely measuring concentrations of inflammatory mediators in body fluids during the development of MS have been largely eliminated by improvements in collection and storage procedures, statistical requirements plus the advent of more sensitive and standardised techniques such as enzyme-linked immunosorbent assay (ELISA) and multiplex technology which includes the electrochemiluminescence-based Meso-Scale Discovery platform and the Lumiex multi-analyte platform systems [114–122]. In addition, there has been a realisation that immune biomarker concentrations during inflammatory conditions such as MS may change with increasing age and independent of disease which necessitates the inclusion of age-matched controls. Also, indicator levels may fluctuate according to circadian and seasonal rhythms [114, 116, 123, 124].

## Criteria for evaluation of the recognised biomarkers of IFA during the pathogenesis of MS

There has been, over the last 2 decades, a determined and progressive approach towards the standardisation of analytical procedures that, in particular, measure the now-recognised biomarkers of IFA. Therefore, a date-dependent boundary has been defined that creates a point from which to begin a detailed and regulated assessment of data investigating the participation of an ongoing low-level inflammation in the pathology of MS. The Review has assessed results from experiments that have determined the circulatory level of each IFA-associated biomarker in MS patients and controls. Specifically, only studies recording significant changes in the concentrations of the individual biomarkers detected in MS patients, compared to controls, have been included in the analysis. Additional criteria for the evaluation and inclusion of quantitative biomarker measurements has endeavoured to exclude analysed samples from MS patients receiving drug therapy and include observations that incorporate comparative control values from subjects with non-inflammatory neurological diseases. Furthermore, the recognised and continuous biological interface between systemic immunity and the CNS has been broadened to include support for a progressive IFA in normal and diseased ageing brain tissue that is also linked to ISC and neurodegenerative events [103, 125]. Therefore, data has been independently considered from sampled blood and cerebrospinal fluid (CSF) to distinguish the representative IFA-associated biomarkers in peripheral and central compartments that denote circulatory and CNS suboptimal inflammation.

### IFA-correlated biomarkers in MS

#### IL-6

The multifunctional cytokine, IL-6, consists of 212 amino acids and a core molecular mass of approximately 20 kilodaltons (kDa). The cytokine is rapidly induced and ubiquitously present following tissue disruption or inflammation resulting from injury or infection which is invariably typified by the detection of upper ng/ml levels in the circulation [126–131]. IL-6 holds an important central position in pivotal immune and inflammatory responses through participation in acute phase reactant production that includes hepatocyte-derived CRP, the regulation of bone marrow-located haematopoiesis and immune cell differentiation which, for example, determines B cell activation, effector T cell development and immunological tolerance. The biological activity of IL-6 is conveyed via an intracellular signalling system that involves membrane-bound and soluble IL-6 receptor occupancy by the cytokine followed by interaction with the β-receptor glycoprotein130 transducer [132]. Various investigations have implicated the cytokine in the pathogenesis of cancers and chronic autoimmune diseases including RA and MS and IL-6 blockade has proved highly effective in the treatment of inflammatory- and immunological-based diseases [133–135].

#### Systemic IL-6 concentrations

There are many reports of raised IL-6 levels in the sera of MS patients despite studies to the contrary describing no quantitative differences from control values or low level detection together with measurement of the cytokine in only a small percentage of patients [136–142]. Indeed, significantly high levels of IL-6, in the pg/ml range, have been confirmed in sera from MS patients with relapsing-remitting disease, during relapse and in association with established neurological disability [143–146]. In addition, patients with progressive but clinically stable MS had raised serum IL-6 concentrations which contrasted with lower levels when disease was ongoing [147]. Moreover, gender differences have been noted with higher concentrations recorded in samples from diagnosed females [148, 149]. Also, increased quantities of IL-6 have been verified in sera from MS patients experiencing depressive illness [150, 151].

#### CSF IL-6 levels

Several studies assaying IL-6 in CSF have reported undetectable levels, low concentrations or no measurable differences compared to controls [136, 138, 140, 141, 152–154]. Also, there are reports that the cytokine is present in only a small number of MS patients or lower and occasionally reduced when compared to other neurological diseases [120, 137, 142, 155]. In contrast, many investigations have recorded raised IL-6 CSF levels in MS patients and, more strikingly, when the severity of neurological disease has been defined [145]. For example, the relapse phase of MS is characterised by elevated quantities of the cytokine [156–159]. L-6 is also higher in clinically evident but stable disease and increases coincide with new pathology in the CNS [10, 161]. Moreover, there appears to be a temporal relationship between MS and IL-6 with high concentrations detected during the progression or in association with the duration of the condition and in protracted undiagnosed disease [143, 161, 162]. Therefore, the analysis suggests IL-6 levels in MS patients with undefined neurological symptoms are similar to control values but reveals that concentrations become noticeably altered when matched with the category of neurological disability. Furthermore, the finding may be relevant to revealing disparities in the levels of the other defined biomarkers of IFA and, indeed, any inflammatory mediators identified as important in the pathogenesis MS.

#### CRP

The pentraxin, CRP is one of several acute phase reactants that are produced by hepatic tissues after specific receptor activation by a range of stimuli including the cytokines IL-1 and IL-6 in response to injury, infection or inflammation [163–165]. The protein, so named because of the ability to react with the somatic c-polysaccharide of *Streptococcus pneumoniae*, exists *in vivo* in pentameric and monomeric forms. Normal circulatory levels of CRP, determined by increasingly sensitive techniques, are less than 1μg/ml but may reach levels in excess of 1mg/ml during inflammatory episodes [118, 166]. A number of functions have been assigned to CRP including a regulatory role in inflammation through activation of the complement pathway and via Fc receptor occupation that is essential for immune defences [167]. Interestingly, low levels of the acute phase reactant that exceed baseline values may be associated with the biological ageing process [168] and a recent study by Jimenez et al [169] has suggested an important role for the protein in regulating age-linked alterations to adaptive immunity and immune tolerance.

#### Circulatory CRP concentrations

Many studies have monitored circulatory CRP levels in the various clinical sub-types of MS with the typical and defining pathophysiology at the time of sampling. For example, plasma and serum concentrations of the protein were measured in clinically undifferentiated MS patients and over the course of relapsing-remitting disease, during a remission plus the progressive form of the disorder and were within normal limits [170, 171]. In contrast, early and many more recent studies have detected increased serum levels of the protein in relapsing-remitting MS patients, during a clinical remission, through primary and secondary progressive disease and also in pregnant women with the disorder [172–179]. In addition, noticeably higher concentrations of CRP were detected at the onset of disease in young subjects compared to individuals showing initial symptoms at an older age [180]. Also, supplementary work has suggested an association between circulatory CRP levels and the inflammatory pathology plus blood-brain barrier disturbances characteristic of MS [181].

#### CSF CRP levels

In contrast to the studies measuring systemic amounts of CRP in MS patients there is a distinct absence of data relating to levels in CSF but a singular increase has been confirmed in samples taken during active disease [179]. Notably, the appraised levels of CRP in systemic and CNS-related samples were in the pg/ml range and therefore, not symptomatic of acute inflammatory events or accompanying infections but indicative of an underlying and ongoing inflammation.

#### TNF-α

Cytotoxicity to tumor cell lines was an early recorded activity of the cytokine TNF-α, formerly known as cachectin, that is produced by a variety of haemopoietic cells including monocytes, macrophages, T lymphocytes and B cells and, in the CNS, by glial cells and neurons [182]. TNF-α, has a diverse and sometimes opposing range of biological actions that include the maintenance of homeostasis and the development of disease pathogenesis that, in particular, feature the immune system and inflammatory processes. The cytokine is synthesised as a bioactive 26 kD transmembrane protein precursor that can be modified, by TNF-α-converting enzyme, to generate a 17-kD soluble form that acts locally or at sites distant from production [183]. The biological roles of TNF-α are primarily conveyed through transmembrane protein receptors, designated TNF-receptor 1 and TNF-receptor 2, that reside on numerous types of nucleated cells with expression of the latter receptor more regulated and restricted to immune cells, the endothelium and neurons. Binding of TNF-α to TNF-receptors 1 and 2 activates complex signalling cascades that have profound effects on cell function to regulate proliferation, survival and programmed death. Normal systemic levels of TNF-α are typically in the low pg/ml range but frequently reach considerably higher concentrations in inflammatory and infectious diseases [184].

#### Circulatory TNF-α levels

There are a few reports of no differences in serum TNF-α concentrations between MS patients with newly diagnosed or long-term disease and controls [138, 141, 185, 186] but data from an overwhelming number of studies show large differences ranging from pg/ml to ng/ml levels of the predominantly pro-inflammatory cytokine. For example, raised amounts of TNF-α were detected in plasma and sera from MS patients with established disease [120, 139, 146, 171, 187–192] and in clinical sub-types of the condition. Indeed, levels were increased in relapsing-remitting and secondary progressive MS and were particularly high during primary progressive disease compared to the other defined categories [176, 180, 193, 194]. Moreover, elevated concentrations were detected during relapse and were sometimes in excess of values in the remission phase of disease which have also been shown to vary compared to controls [195–199]. In addition, increased amounts of TNF-α in plasma and serum samples have been found to correlate with clinical episodes of disease, an increase in neurological deficits and enhanced lesion load particularly in patients with primary progressive MS [144, 147, 179, 194, 200, 201].

#### CSF TNF-α concentrations

Quantitation of TNF-α in CSF sampled from verified but clinically undifferentiated cases of MS and newly diagnosed patients have confirmed values similar to controls [120, 137, 138, 141, 145, 152, 186]. In contrast, several studies have identified raised ng/ml levels of TNF-α in CSF from patients with established MS [103, 154, 188]. Moreover, concentrations of the cytokine were high in progressive MS, [202, 203] during clinically apparent disease [179, 195] following initial diagnosis and later development of disease [204]. CSF TNF-α concentrations were also increased in relapsing and secondary progressive disease compared to levels in the remission phase [187].

#### IL-1Ra

Initially, the 17 kDa polypeptide IL-1Ra was recognised as a suppressor factor of IL-1-mediated thymocyte proliferation and subsequent investigations revealed an overall ability to curtail IL-1 bioactivity [205]. IL-1Ra competitively inhibits the ubiquitous cellular receptor IL-1R type 1 by competing with IL-1α and IL-1β to block the signalling processes leading to the release of and interaction between mediators that determine systemic inflammation [206]. The anti-inflammatory properties of IL-1Ra have been successfully duplicated in a human recombinant, non-glycosylated version, expressed in *Escherichia coli*, and named Anakinra which has been approved for the treatment of arthritis and other members of the arthritidies family [207].

#### Systemic and CSF IL-1Ra levels

There is minimal information on serum and CSF concentrations of IL-1Ra in MS patients not receiving therapy. Heesen et al [208] recorded high serum levels, compared to controls, in active disease and during clinically stable episodes. Values were also increased, to upper pg/ml concentrations, in progressive MS as opposed to patients with relapsing-remitting disease. In contrast, later studies in a sample of clinically undefined MS patients found amounts in serum were similar to controls. However, the investigation did detect significantly raised quantities in CSF taken from the patients [209]

#### IL-10

Down-regulation of the inflammatory process is a major property of the cytokine IL-10, originally known as cytokine synthesis inhibitory factor, which is released from a variety of peripheral immune cells, including macrophages, B cells and T cell subsets, and expressed by glia and neurons in the CNS [210]. IL-10 exists as a 35 kD molecule and exerts anti-inflammatory effects via occupation of an individualised cellular transmembrane glycoprotein receptor complex that activates to inhibit several inflammatory-linked cytokines, including TNF-α and IL-6, and stimulates the augmented release of IL-Ra [211]. In addition, IL-10 receptor activation disrupts antigen presentation by reducing cell surface expression of the MHC plus co-stimulatory and adhesion molecules together with CD4^+^ T cell proliferation.

#### Systemic IL-10 levels

Several reports have found the levels of IL-10 in serum and plasma from newly diagnosed MS patients and those with more established disease to be within the range of controls [140, 141, 145, 192]. In contrast, there are various studies that have described significantly increased concentrations of IL-10, at pg/ml levels, in blood and serum sampled from MS patients and, in particular, taken during remission, and in conjunction with the neurological changes accompanying relapsing-remitting and progressive disease [139, 147, 177, 191, 196, 212].

#### CSF IL-10 concentrations

Limited early and later work measuring IL-10 concentrations in CSF from MS patients found no differences compared to controls [141, 152, 213]. However, a complementary series of studies found an opposing profile which revealed elevated IL-10 levels, at pg/ml concentrations, in CSF from MS patients including samples obtained during relapsing-remitting disease [145, 154]. Also, increased amounts of the cytokine were detected with recent diagnosis and during apparent ongoing neurological symptoms [103, 179, 209].

## Summary and critique of biomarker-based evidence for IFA during MS

A significant change in the systemic levels of CRP, IL-6, TNF-α, IL-1Ra and IL-10 are defined indicators of IFA. The Review has established, from joint appraisal of the literature, that the majority of studies designed to measure concentrations of the classified IFA-associated biomarkers in sampled blood and CSF from MS phenotypes have recorded significant alterations compared to control values. Noteworthy, the reports describing significant changes for each IFA-associated biomarker and expressed as a percentage of the total number of studies (Table 1) excludes investigations that detected noticeably increased, but not significant, circulating levels of some biomarkers in samples from MS patients compared to controls. Interestingly, and despite the absence of significant variation, the sampling of MS patients with raised biomarker levels may have coincided with an activated state of IFA that had either not optimised or had peaked and was in decline. In addition, the low-level increase of each biomarker is representative of a sub-optimal inflammatory response and not an acute inflammatory effect where recorded levels of reactants, including CRP, IL-6 and TNF-α, are typically and markedly raised [214, 215]. Therefore, the Review has evaluated and presented sufficient collective evidence to indicate the occurrence of IFA over the course of MS and, more specifically, during typical clinically-distinct phases of the disease.

**Table 1:** The number of studies and the percentage (%) detecting significant change in systemic levels of IFA-related biomarkers.

IFA biomarker	Number of studies/% detecting significant change	
	Serum/Plasma	CSF
IL-6	18/60	21/52
CRP	14/71	^a^
TNF-α	30/87	16/56
IL-1Ra	2/50	2/50
IL-10	10/60	8/63

^a^ One study [179] confirmed a raised level of CRP that was not significantly different from control values.

Evaluation of the studies also revealed the intricacies of biomarker quantitation in an age-related disease that includes several clinical categories and a protracted pathophysiology. The mechanisms governing acute phase reactant and cytokine production and availability during inflammatory responses and at sampling time are complex with a reliance on mutual interaction and a profile of transitory release [155, 216, 217]. Collective appraisal of the data also suggests an interconnected regulation of the biomarkers that includes an inverse relationship between the levels of pro- and anti-inflammatory mediators. In addition, and with particular reference to MS, the physiological distribution of the inflammatory mediators is often restricted which hinders identification and, despite upregulation of the analogous receptors, may not be generated in sufficient amounts for detection [218, 219]. Also, there is an emerging opinion that an inherent IFA process may impinge on systems that determine inflammatory indicator levels and obscure differences between healthy and disease states. For instance, Hu et al [103] found the CSF content of TNF-α in healthy subjects increased linearly with age. In contrast, IL-10 concentrations were higher in samples from young and old individuals, compared to a middle-aged group, which generated a non-linear U-shaped distribution of data with a lower detection at the centre of the range.

The biological and disease-related dynamics associated with the measurement of inflammatory mediators may help to explain the disparity between investigations that detected normal and elevated levels of the IFA biomarkers. In addition, differences in experimental design and methodology may contribute to variances between groups. For example, and collectively, much of the work incorporated small and unequal sample sizes together with asymmetrically distributed data that would alter statistical power, as acknowledged by Wullschleger et al [155], who measured IL-6 concentrations in CSF taken from patients with MS and other neurological inflammatory diseases. In addition, the studies detailed measurements obtained from several contrasting techniques including ELISA, fluorescent-activated cell sorting analysis and multiplex systems with variable limits of detection for each biomarker.

## Conclusions and guidelines

The Review has presented compelling evidence from a range of quantitative inflammatory-associated biomarker studies that supports the presence of IFA during the pathogenesis of MS. Moreover, the occurrence of IFA is confirmed in all clinical sub-types and neurological phases of the disease. Notably, there is general agreement that the variable chronic inflammation, which declines with age and disease duration, is confined to the central nervous system CNS [60, 220]. Therefore, the IFA-related biomarkers detected at significant concentrations in the majority of serum-derived samples from MS patients probably originate from circulatory inflammatory cell components than constituents of the CNS. Also, the biomarker-associated systemically-located inflammation may be secondary to the chronic and primary facilitator of disease in the CNS. Indeed, the enduring CNS-restricted inflammation has been observed in acute white matter lesions and as part of a reactive process which may be ancillary to a principle neurodegenerative process [221, 222]. Consequently, the marginal majority of significantly raised IFA-associated biomarkers in CSF from MS patients may reflect the sustained presence of a sub-optimal inflammation that subtly drives the disease particularly in the established and increasingly progressive stages that develop with increasing age.

Previously discussed and referenced findings have acknowledged that the chronic systemic sub-maximal inflammation typifying IFA is coincidental with ISC and, in particular, linked with a premature ageing of the adaptive immune system which impacts on the efficiency of immunological tolerance. Earlier observations by us confirmed ISC and demonstrated an untimely ageing of the immune system in MS which was suggested causal to the recognised increase in the incidence and prevalence of the disease. Therefore, the verified occurrence of IFA during MS accompanies an age-dependent remodelling of the immune system that provides an interactive relationship through which unfavourably-timed alterations to immune ageing and immunological tolerance may proceed.

Further specific biomarker-related work is required to strengthen and develop the concept of IFA ongoing in MS and define the relationship with the processes of ISC and immune tolerance. A guide to prospective studies is offered through a variety of analogous investigations that provide valuable information on putative intrinsic and extrinsic stressors triggering IFA through diverse actions on the innate and adaptive immune system [223–228]. Moreover, and as illustrated in immune-related, age-associated comparative work, there is an important requirement for longitudinal IFA biomarker studies to assess indicator levels with increasing age in healthy controls and patients with MS and other neurological diseases [229–231]. The comparative investigations also promote understanding of the mechanisms that challenge the dynamics of age-related immune remodelling and disrupt immune tolerance which is prominent in autoimmune-based diseases such as MS.

## Data Availability

Not applicable.

## Abbreviations

CD: Cluster of differentiation
CNS: Central nervous system
CRP: C-reactive protein
CSF: Cerebrospinal fluid
ELISA: Enzyme-linked immunosorbent assay
GARP: Glycoprotein A repetition predominant
GHO: Global health observatory
IFA: Inflammageing
IL: Interleukin
ISC: Immunosenescence
kD: Kilodalton
MHC: Major histocompatibility complex
MS: Multiple sclerosis
Ra: Receptor antagonist
RA: Rheumatoid arthritis
SASP: Senescence-associated secretory phenotype
SLE: Systemic lupus erythematosus
TNF-α: Tumor necrosis factor-α
